# Temporal and Spatial Dynamics in the Regulation of Myocardial Metabolism During the Ischemia-Reperfusion Process

**DOI:** 10.3390/ijms26188820

**Published:** 2025-09-10

**Authors:** Elena de Dios, Maria J. Forteza, Nerea Perez-Sole, Tamara Molina-Garcia, Jose Gavara, Victor Marcos-Garces, Manuel Jimenez-Navarro, Amparo Ruiz-Sauri, Cesar Rios-Navarro, Vicente Bodi

**Affiliations:** 1 Centro de Investigación Biomédica en Red–Cardiovascular, 28029 Madrid, Spain; elenaddll@gmail.com (E.d.D.); neere_8@hotmail.com (N.P.-S.); jose.gavara@outlook.es (J.G.); marcos_vic@gva.es (V.M.-G.); mjimeneznavarro@uma.es (M.J.-N.); ruizsa@uv.es (A.R.-S.); 2 Cardiovascular Medicine Unit, Center of Molecular Medicine, Department of Medicine, Karolinska Institutet, Karolinska University Hospital, 17176 Stockholm, Sweden; mjforteza@gmail.com; 3 Medicine Department, Faculty of Medicine, University of Valencia, 46010 Valencia, Spain; tamara5mg1999@gmail.com; 4 INCLIVA Biomedical Research Institute, 46010 Valencia, Spain; 5 Heart Institute, Hospital Universitari Germans Trias i Pujol, 08916 Badalona, Spain; 6 Cardiology Department, Hospital Clínico Universitario, 46010 Valencia, Spain; 7 Cardiology Department, Hospital Universitario Virgen de la Victoria, 29010 Malaga, Spain; 8 Medicine Department, Faculty of Medicine, University of Malaga, 29010 Malaga, Spain; 9 Instituto de Investigación Biomédica de Málaga y Plataforma en Nanomedicina (IBIMA Plataforma Bionand), 29590 Malaga, Spain; 10 Pathology Department, Faculty of Medicine, University of Valencia, 46010 Valencia, Spain

**Keywords:** cardiac metabolism, myocardial infarction, glycolysis, beta-oxidation

## Abstract

Although cardiac metabolic adaptation has been observed in response to the ischemia–reperfusion, the specific temporal and spatial changes occurring in the main regulators of myocardial glucolipid metabolism in the infarcted heart have not been fully characterized. Myocardial infarction (MI) was induced in female swine by transient coronary occlusion. The study design consisted of one control and four MI groups (no reperfusion, 1 min, 1 week, and 1 month after reperfusion). Metabolites obtained from the coronary sinus were determined at baseline, during ischemia, and after coronary reperfusion. mRNA expression of genes related to beta-oxidation and glucose transport were quantified in the five experimental groups and in three myocardial regions (infarcted, adjacent, and remote). In the coronary sinus, reduced glucose and increased lactate levels were detected during ischemia and soon after reperfusion. However, non-esterified fatty acids increased during reperfusion. A general upregulation of genes implicated in glycolysis and beta-oxidation occurred during ischemia and few minutes after reperfusion. Contrarily, heightened mRNA expression of glucose transporters and decay in regulators of beta-oxidation were observed one week after coronary reperfusion. Glycolysis and beta-oxidation are activated during ischemia and few minutes after coronary reopening, while a shift from beta-oxidation to glycolysis is evidenced a few days afterwards.

## 1. Introduction

The adult heart normally consumes fatty acids (FA) for its metabolism, but cardiac tissue has developed a delicate adaptation program in response to hypoxic stimuli in which glucose is used preferentially on FA to maintain systolic function and satisfy energy demand [1,2,3]. Although FA metabolism is the main producer of ATP in the heart, glucose oxidation consumes less oxygen, making it more efficient in hypoxic situations [4,5,6]. During ischemia, oxygen decreases, glucose oxidation slows down, and glycogen is degraded to feed glycolysis, which in the absence of oxygen has lactate as a final product.

In the myocardium, proliferator-activated receptor-γ coactivator-1α (PGC-1α) plays a pivotal role in the physiologic response to hypoxia [7,8]. In cardiomyocytes, PGC-1α is considered a master metabolic regulator for its co-activation of peroxisome proliferator-activated receptor-α (PPARα) and estrogen-related receptor-α (ERRα) [7,8,9]. The interrelationship between PGC-1α and PPARα plays an important role in regulating the expression of enzymes involved in FA oxidation and uptake pathways and may be involved in mitochondrial biogenesis regulation [8,9,10].

Glucose transport into cardiomyocytes is regulated by the transmembrane glucose gradient and the content of glucose transporters in the sarcolemma [mainly glucose transporter (GLUT)-4, and to a lesser extent GLUT-1] [4,5,6]. There is a translocation of glucose transporters from intracellular vesicles to the sarcolemma in response to different stimuli, including myocardial ischemia [5,6]. Consequently, the membrane capacitance for glucose transport and the rate of glucose uptake is increased.

Enhancing understanding of the mechanisms that regulate cardiac metabolism following myocardial infarction (MI) to maintain systolic function could be highly instrumental for developing new therapeutic interventions focused on modulating and optimizing the energy substrate [11]. In the current study, we examined temporal changes in circulating metabolites together with gene expression patterns related to substrate metabolism, to provide a comprehensive overview of myocardial metabolic adaptation in response to ischemia–reperfusion injury.

## 2. Results

Seventeen juvenile domestic female pigs weighing 25–30 kg were utilized. Two of these died during balloon inflation due to refractory ventricular fibrillation, while electrical ventricular defibrillation was needed in a further three pigs during left anterior descending (LAD) occlusion. No significant complications were recorded over the reperfusion period. LAD patency was confirmed in all pigs before sacrifice. Successful experiments were divided into five groups: control group (*n* = 3), no reperfusion (*n* = 3), 1 min (*n* = 3), 1 week (*n* = 3) and 1 month (*n* = 3) after reperfusion.

Macroscopic analysis of hearts submitted to MI was conducted. According to our findings, no differences were observed in LAD-perfused area or infarct area across the groups (Table 1). Indeed, myocardial wall thickness was measured in three distinct regions (infarct, adjacent, and remote myocardium) and no changes were evidenced among the groups (Table 1).

### 2.1. Concentration of Metabolites in the Coronary Sinus Blood

**Figure 1:**
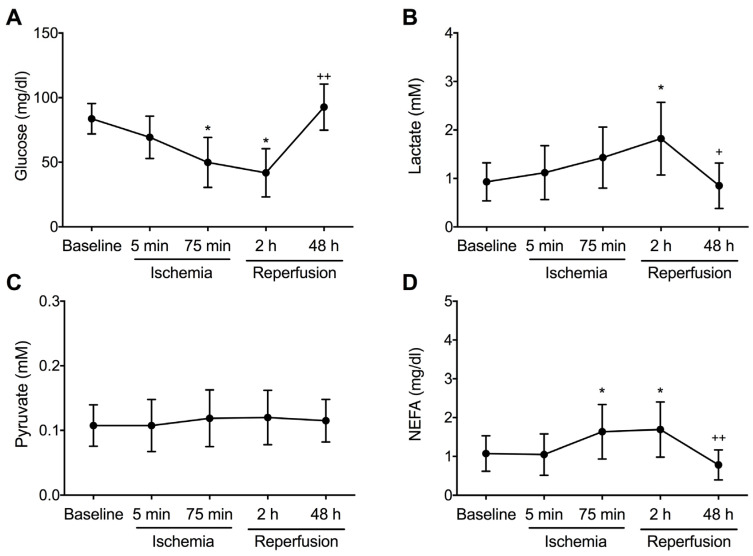
Temporal changes in the levels of different metabolites determined in the coronary sinus. Serum levels were isolated at baseline (before coronary occlusion), during ischemia (5 min and 75 min after balloon inflation) and after reperfusion (2 h and 2 days after balloon deflation) from the coronary sinus. Levels of glucose (**A**), lactate (**B**), pyruvate (**C**), and non-esterified fatty acids (NEFA, (**D)**) were determined at different timepoints after ischemia–reperfusion injury. Data (mean ± SD, *n* ≥ 6) were analyzed by one-way ANOVA analysis followed by Bonferroni test. * *p* < 0.05 vs. control group; ^+^ *p* < 0.05, ^++^ *p* < 0.01 vs. 2 h reperfusion.

To investigate temporal changes in levels of different metabolites [glucose, lactate, pyruvate, and non-esterified fatty acid (NEFA)], measurements were repeated at predefined timepoints: at baseline (before coronary occlusion), during ischemia (5 min and 75 min after balloon inflation) and after reperfusion (2 h and 2 days after balloon deflation). Circulating glucose levels decreased during ischemia and after reperfusion, reaching their lowest point at 2 h (42 mg/dL), whereas there was a progressive increase in circulating levels of lactate after reperfusion, peaking 2 h after revascularization (1.8 mM). No temporal changes in pyruvate concentration were observed). Finally, NEFA levels increased significantly 75 min after ischemia onset (1.6 mg/dL) and remained significantly elevated until 2 h post-reperfusion (1.7 mg/dL). The concentration of glucose, lactate, and NEFA returned to baseline levels 48 h after coronary occlusion (Figure 1).

These data suggest that glucose consumption is highest soon after coronary reopening, as reflected by the reduction in glucose levels and accumulation of lactate and NEFA 2 h after reperfusion.

### 2.2. Gene Expression Dynamics in FA Metabolism and Glucose Uptake Regulation During Ischemia and Soon After Reperfusion

To elucidate changes in circulating metabolite levels, we quantified the gene expression of primary molecules involved in regulating FA metabolism (PGC-1α, PPARα, and ERRα) and glucose uptake (GLUT-1 and GLUT-4) during ischemia and immediately after reperfusion in the infarcted, adjacent, and remote regions.

**Figure 2:**
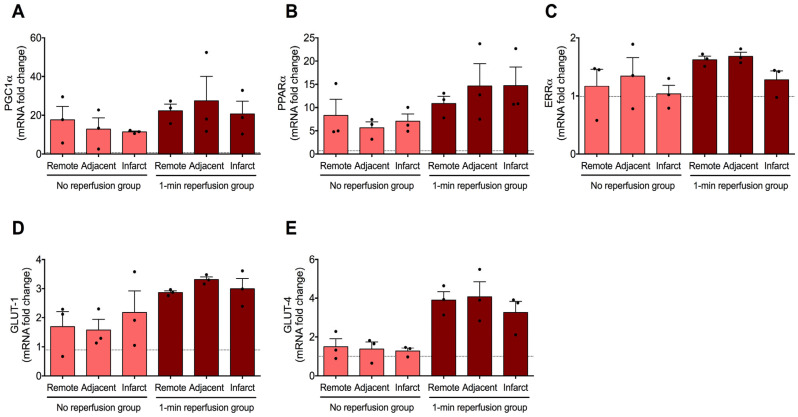
mRNA expression of key genes implicated in the metabolism of fatty acid and glucose intake in infarcted, adjacent, and remote myocardium isolated after ischemia and soon after reperfusion. The gene expression of peroxisome proliferator-activated receptor-γ coactivator-1α (PGC-1α, (**A**)), peroxisome proliferator-activated receptor-α (PPARα, (**B**)), estrogen-related receptor-α (ERRα, (**C**)), glucose transporter 1 (GLUT-1, (**D**)), and glucose transporter 4 (GLUT-4, (**E**)) was determined in the infarcted, adjacent, and remote myocardium from the no reperfusion group (90 min of ischemia without coronary reopening) and 1 min reperfusion group (90 min of ischemia followed by 1 min of reperfusion) compared to control group (dotted line). Data were expressed as mean ± SD (*n* = 3). Black dots represent raw values.

Regarding FA metabolism, there was a notable increase in PGC-1α and PPARα gene expression 90 min after ischemia onset compared to baseline levels. These changes were observed in the myocardium subjected to ischemia (infarcted and adjacent areas) and in the remote myocardium, where blood perfusion was completely preserved. This tendency persisted immediately after coronary reperfusion (Figure 2A,B). Contrarily, the mRNA expression of ERRα was upregulated only after revascularization, but not during myocardial ischemia (Figure 2C).

Gene expression analysis of glucose transporters revealed transcriptomic levels of both molecules similar to those in the control group during ischemia. However, a rise in mRNA levels of GLUT-1 and GLUT-4 was detected immediately after revascularization. This tendency was similar in the three evaluated regions (infarcted, adjacent, and remote) (Figure 2D,E, respectively).

These results suggest that molecules involved in regulating FA metabolism were overexpressed during myocardial ischemia. In contrast, the gene levels of glucose transporters increased after coronary reperfusion. These variations occurred throughout the entire myocardium, as transcriptional alterations were similar regardless of whether myocardial perfusion was blocked (infarcted and adjacent areas) or preserved (remote area).

### 2.3. Gene Expression Changes in Molecules Implicated in FA Metabolism and Glucose Uptake in Subacute and Chronic Phases After Coronary Reperfusion

To further explore alterations in the main regulators of FA metabolism and glucose transporters, their gene levels were quantified one week and one month after coronary reperfusion. In line with previous analyses, these alterations were determined in the infarcted, adjacent, and remote regions.

**Figure 3:**
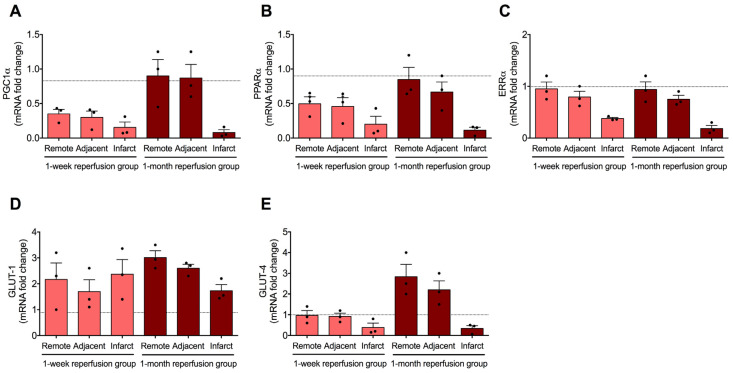
mRNA expression of key genes implicated in the metabolism of fatty acid and glucose intake in infarcted, adjacent, and remote myocardium isolated one week and one month after coronary reperfusion. The gene expression of peroxisome proliferator-activated receptor-γ coactivator-1α (PGC-1α, (**A**)), peroxisome proliferator-activated receptor-α (PPARα, (**B**)), estrogen-related receptor-α (ERRα, (**C**)), glucose transporter 1 (GLUT-1, (**D**)), and glucose transporter 4 (GLUT-4, (**E**)) was determined in the infarcted, adjacent, and remote myocardium from the 1 week and 1 month reperfusion groups compared to the control group (dotted line). Data were expressed as mean ± SD (*n* = 3). Black dots represent raw data.

According to our data, all three FA metabolism regulators exhibited diminished gene expression in the infarcted myocardium isolated one week and one month after MI induction. The myocardium from adjacent and remote regions showed decreased transcriptomic levels of PGC-1α and PPARα, but not ERRα, in the one-week reperfusion group (Figure 3A–C). Lastly, one month after coronary revascularization, the mRNA levels of the main regulators of FA metabolism were similar to control myocardium in the adjacent and remote areas. Overall, PGC-1α, PPARα, and ERRα exhibited a bimodal pattern: rapid gene overexpression in the whole heart during myocardial ischemia and the initial moments of reperfusion, contrasting with decreased levels in the infarcted area at chronic phases, returning to control levels in the adjacent and remote regions.

Regarding glucose transporters, GLUT-1 gene expression was heightened in all three regions from the first week post-MI onward (Figure 3D). Contrariwise, GLUT-4 mRNA levels decreased in infarcted tissue, while the adjacent and remote zones exhibited GLUT-4 overexpression one month after coronary reopening (Figure 3E). These results therefore demonstrated overexpression of GLUT-1 in the heart overall from the initial minutes after coronary reperfusion to chronic phases. Conversely, GLUT-4 levels were elevated immediately after coronary reperfusion but remained augmented in the adjacent and remote areas, while they were reduced in the infarcted area in the one-month reperfusion group.

## 3. Discussion

### 3.1. Cardiac Metabolism Under Physiological and Pathological Conditions

Under normoxic conditions, FA and glucose are the primary sources of energy in the heart [1,2,3] and impairments in glucolipid metabolism can rapidly lead to adverse ventricular remodeling and abnormal cardiac contractility [12,13,14].

PGC-1α is considered a key regulator of cellular metabolism and energy homeostasis. Specifically, PCG-1α regulates genes implicated in the utilization of FA and glucose as energetic substrates and in mitochondrial biogenesis, which is essential for maintaining adequate ATP production through oxidative phosphorylation [7,8]. Several studies indicate that PGC-1α disruption can lead to heart failure and cardiac dysfunction, making it a prime target for potential therapeutic interventions aimed at enhancing cardiac contractility [13,15]. In cardiomyocytes, PGC-1α modulates cardiac metabolism via PPARα and ERRα co-activation. PPARα participates in promoting FA uptake and oxidation by regulating transport proteins, mitochondrial β-oxidation enzymes, and electron transport chain components. ERRα maintains mitochondrial activity and oxidative phosphorylation [7,8,9]. Changes in PPARα and ERRα signaling are linked to a metabolic shift from FA oxidation to glucose metabolism, mitochondrial dysfunction, and reduced ATP production [12,16].

Regarding glucose uptake, cardiomyocytes primarily utilize GLUT-1 and GLUT-4. GLUT-1 is located in the sarcolemma under physiologic conditions and provides a constant, low-level supply of glucose. In contrast, GLUT-4 is the predominant isoform in adult cardiomyocytes and translocates from intracellular vesicles to cell membrane in response to various stimuli, including ischemia or catecholamines [4,5,6]. Enhanced understanding of the mechanisms that regulate cardiac metabolism in physiological and pathological situations could play a key role in developing new therapeutic interventions focused on modulating and optimizing the energy substrate.

### 3.2. Changes in Circulating Metabolites After Ischemia–Reperfusion Injury

MI causes an abrupt complete occlusion of a coronary artery, leading to a lack of oxygen and nutrients to the downstream myocardium [17]. As a consequence, cardiac metabolism is shifted as reflected by temporal changes in circulating metabolites in the coronary sinus [18]. In line with previous investigations reporting that reperfusion induces acute metabolic stress [11], our current data point to a notable decay in circulating glucose levels two hours after reperfusion, suggesting a peak in glucose consumption during the early reperfusion phase. These findings align with the increased expression of glucose transporters GLUT-1 and GLUT-4 after coronary reopening, which facilitate enhanced myocardial glucose uptake. Collectively, this likely reflects a surge in metabolic demand by ischemic myocardial tissue attempting to maintain systolic function, consistent with reports indicating augmented glucose uptake and glycolytic activity after ischemia–reperfusion injury [11].

Parallel to the drop in glucose, a significant augmentation in lactate levels is also observed, peaking at two hours post reperfusion. This indicates the activation of anaerobic metabolism and insufficient mitochondrial oxidative capacity soon after coronary reopening. This lactate accumulation corroborates earlier observations of metabolic inflexibility and hypoxia-induced glycolysis during early reperfusion [19].

Our data also evidenced unchanged pyruvate levels throughout the ischemia–reperfusion timeline. This might be due to rapid turnover or equilibrium between production and utilization, which may mask transient fluctuations.

Lastly, quantities of circulating NEFA were significantly heightened before ischemia onset and remained elevated up to two hours post-reperfusion. A recent study concluded that circulating NEFA levels are independently associated with sudden death, probably due to proarrhythmic mechanisms [20]. Indeed, sustained NEFA levels could be related to stress-induced lipolysis and may contribute to lipotoxicity, oxidative stress, and impair cardiac function.

Overall, while reperfusion is crucial for salvaging cardiac tissue, it also triggers oxidative stress, mitochondrial dysfunction, and metabolic alterations that impair cardiac function. Once the initial stress response subsides, however glucose, lactate, and NEFA levels returned to baseline, thus indicating metabolic recovery.

### 3.3. Temporal and Spatial Transcriptomic Alterations Related to Metabolic Adaptation After Ischemia and Reperfusion Injury

To further understand the changes in circulating metabolites, we also evaluated the main genes implicated in cardiac metabolism regulation in response to substrate availability and oxygen tension following ischemia–reperfusion injury.

During ischemia, we detected increased mRNA expression of PGC-1α, PPARα, and ERRα. Even though FA oxidation is oxygen-intensive, this upregulation could represent a maladaptive response of cardiac metabolism programming, potentially contributing to inefficient ATP production and lipotoxicity under oxygen deprivation. While FA remains the main energy substrate in the ischemic myocardium, respiration and oxidative phosphorylation functions of mitochondria are substantially impaired during ischemia [1,2,3]. There is evidence that the number of mitochondria is augmented in acute myocardial ischemia, which might be a compensatory response to acute ischemia and hypoxia [19]. In this line, prior studies have demonstrated that chronic activation of PPARα can impair glucose utilization and exacerbate ischemic injury, despite its role in enhancing FA oxidation under normal conditions [21]. Indeed, in preclinical models of MI, inhibiting FA oxidation via small molecular inhibitors of enzymes participating in FA transport and metabolism significantly reduces infarct extent and improves systolic function [22].

Regarding glucose uptake, GLUT-1 and GLUT-4 overexpression after coronary reperfusion indicates a shift towards increased glucose utilization, which is metabolically advantageous since glucose oxidation is more oxygen-efficient than FA. The overrepresentation of both glucose transporters could be driven by hypoxia-inducible and insulin-mediated pathways, which are activated during ischemia and early reperfusion [23,24]. This might be a compensatory mechanism aimed at restoring myocardial energetics and enhancing recovery following ischemic insult.

In terms of spatial distribution, these transcriptional changes were detected uniformly across the three evaluated myocardial areas (infarcted, adjacent, and remote), thus indicating a global rather than a localized myocardial response. It implies that even remote areas, not directly affected by the lack of oxygen and nutrient supply, undergo metabolic reprogramming. These results are in line with a previous manuscript reporting an increase in GLUT-1 in remote myocardium as early as 6 h after coronary occlusion in dogs [25]. Consequently, the resulting activation of systemic regulatory mechanisms, possibly mediated by neurohormonal responses or inflammatory signals, might orchestrate the modulation of gene expression across the entire heart during ischemia and post-reperfusion. These results also indicate that interventions may need to involve the whole heart even when damage appears localized.

In summary, our data indicate a coordinated temporal and spatial metabolic response in the entire heart during ischemia and reperfusion injury. Initially, heightened mRNA levels of genes promoting FA oxidation during ischemia can be observed, probably as a stress-induced compensatory mechanism. Upon reperfusion, a metabolic shift towards the intake of glucose is noticed, as evidenced by elevated glucose transporter gene expression and corresponding decay in circulating glucose levels.

### 3.4. Myocardial Glucolipid Metabolism at Advanced Phases After Coronary Reperfusion

To detect whether this metabolic profile persists along time after reperfusion, we quantified the expression of these genes at subacute (one week) and chronic (one month) phases after coronary reopening. According to our data, PGC-1α and PPARα expression is reduced in adjacent and remote myocardium, while the three regulators of FA metabolism (including ERRα) are downregulated in the infarcted tissue. By one month, this pattern persists only in the infarcted myocardium, whereas mRNA levels in adjacent and remote zones return to baseline. These results indicate a region-specific adaptation whereby viable myocardium restores oxidative capacity, while the fibrotic scar displays a different metabolic competency.

This bimodal pattern, as reflected by an early whole-heart upregulation followed by selected chronic downregulation of key regulators of FA metabolism, highlights the complexity of cardiac metabolic remodeling post MI. In the infarcted myocardium, these alterations could also be explained by several factors, including altered cellular composition (inflammatory cells in the subacute and myofibroblasts in the chronic phases post MI), reduced capillary density, massive collagen deposition, and mitochondrial loss in the infarcted region [11,23,26].

In parallel, the transcriptomic levels of glucose transporters also undergo distinct temporal and spatial modulation. Based on our data, the mRNA expression of GLUT-1 is overrepresented in all three cardiac regions immediately after coronary reopening through to at least one month post MI. This prolonged overexpression may reflect protective adaptation to ensure basal glucose availability in stressed tissue.

Conversely, GLUT-4 mRNA levels display a more nuanced profile: acutely upregulated after reperfusion, they become persistently downregulated in the infarcted tissue by one month post reperfusion. Interestingly, GLUT-4 expression in adjacent and remote regions increases over time, probably indicating a compensatory mechanism in viable myocardium to meet increased energetic demands. These results are in line with a previous investigation suggesting that during reperfusion, the gene expression of GLUT-1 mRNA is increased, whereas a decay in GLUT-4 mRNA levels are decreased [27].

Altogether, these findings depict a heart submitted to temporal and regional metabolic reprogramming. The acute phase is characterized by a whole heart increase in glucose uptake (decreased glucose levels, rising circulating lactate, increased mRNA expression of glucose transporters), FA-driven transcriptional responses, and systemic NEFA accumulation. As reperfusion progresses into days and weeks, gene levels diverge by myocardial region, permanently depressed in the infarcted area but recovering to baseline levels in non-fibrotic tissue. Regarding clinical implications, the reduced oxidative and glucose handling capacity in infarcted tissue could hinder regeneration and contractile recovery, while augmented glucose uptake in adjacent and remote areas may represent a target for therapeutic metabolic support.

### 3.5. Limitations of the Study

All animals employed in the present study were female. Sex differences influence the myocardial metabolic response under cardiac stress. For instance, female hearts are reported to show higher myocardial oxygen consumption, enlarged expression of FA metabolism genes, and greater mitochondrial mass in comparison to male myocardial tissue [28,29,30]. However, as far as we are concerned, little is known about how post-MI cardiac metabolism differs with biological sex. Taking all together, our conclusions are limited to differences in the regulation of cardiac metabolism from female swine submitted to MI.

In these studies, cardiac function was not assessed, which would have strengthened the translational value of our results.

## 4. Materials and Methods

This study was approved by the local Animal Care and Use Committee (Reference: 2016/VSC/PEA/00074) and complies with the Guide for the Care and Use of Laboratory animals published by the US National Institutes of Health (NIH Publication No. 85-23, revised 1993), as well as European (2010/63/EC) and national regulations (RD53/2013).

### 4.1. Experimental Protocol

Fifteen juvenile domestic female pigs (*Sus scrofa*) weighing 25–30 kg were used in the experimental study. In line with surgical procedure, animals were mechanically ventilated employing a 50% oxygen/gas mixture, with heart rate, rhythm, and ST-segment changes continuously monitored. We administered intramuscular ketamine (8 mg/kg) and medetomidine (0.1 mg/kg) for sedation and continuous intravenous 10 mg/kg/h infusion of 2% propofol for anesthesia induction. To reduce the possibility of life-threatening arrhythmias, pigs were pre-treated with intravenous amiodarone (300 mg) and lidocaine (30 mg). A 7 Fr sheath was introduced into the right femoral artery to monitor blood pressure and access the LAD coronary artery. A 7 Fr Amplatz Left 0.75 catheter was used to selectively engage the proximal LAD and a standard hydrophilic angioplasty wire was advanced and placed in the distal LAD. A 2.5 mm × 15 mm over-the-wire angioplasty balloon was inflated at 6 atm in the mid LAD distal to the first diagonal branch. Coronary artery occlusion was confirmed by contrast injection and by electrocardiographic ST-segment elevation. Further details regarding the experimental protocol can be consulted elsewhere [23,31,32,33].

### 4.2. Experimental Groups

**Figure 4:**
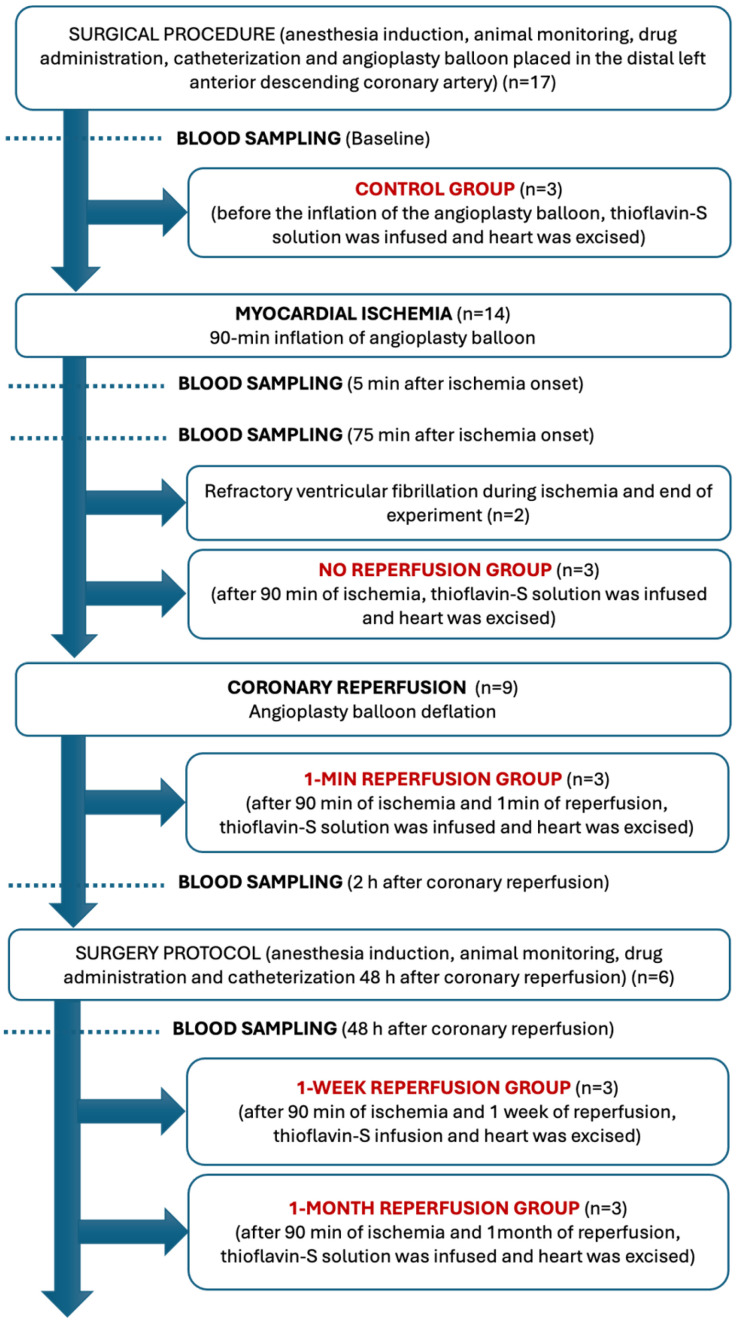
Flow chart of the experimental groups.

One control group and four independent MI experimental groups were formed. The MI groups underwent 90 min occlusion of the mid-LAD artery by the angioplasty balloon, then were stratified as follows: (1) no reperfusion, or (2) 1 min, (3) 1 week and (4) 1 month after reperfusion (Figure 4).

The control group was made up of 3 experiments. In this group, we used the study protocol described above, but without inflating angioplasty balloon and thus ischemia and infarction were not provoked. We selective infused 20 mL of 4% thioflavin-S solution (Sigma Aldrich, St. Louis, MO, USA) into the proximal LAD through the Amplatz Left 0.75 catheter. Hearts were then arrested with potassium chloride (0.9%, 2 mL) and excised [33] (Figure 4).

In the no reperfusion group (*n* = 3), animals underwent 90 min period of ischemia but without reperfusion. In this group of experiments, the balloon was not deflated and, after 90 min of ischemia, 20 mL of 4% thioflavin-S solution was selectively infused into the mid LAD after the first diagonal branch through the lumen of an over-the-wire balloon. Immediately after thioflavin-S administration, hearts were arrested using potassium chloride (0.9%, 2 mL) and then excised [33] (Figure 4).

In the three groups of experiments with reperfusion, the angioplasty balloon was deflated after 90 min of coronary occlusion and restoration of normal coronary flow was documented by angiography. In the 1 min reperfusion group (*n* = 3), 20 mL of 4% thioflavin-S solution was selectively infused into the proximal LAD through the Amplatz Left 0.75 catheter 1 min after balloon deflation, and hearts were arrested with potassium chloride (0.9%, 2 mL) and excised. Swine in the 1-week and 1-mont reperfusion groups were allowed to recover and after 1 week (*n* = 3) or 1 month (*n* = 3), respectively, the same study protocol was followed and 20 mL of 4% thioflavin-S solution was selectively infused into the proximal LAD through the Amplatz Left 0.75 catheter. Hearts were then arrested with potassium chloride (0.9%, 2 mL) and excised [33] (Figure 4).

### 4.3. Macroscopic Analysis of Myocardial Samples

**Figure 5:**
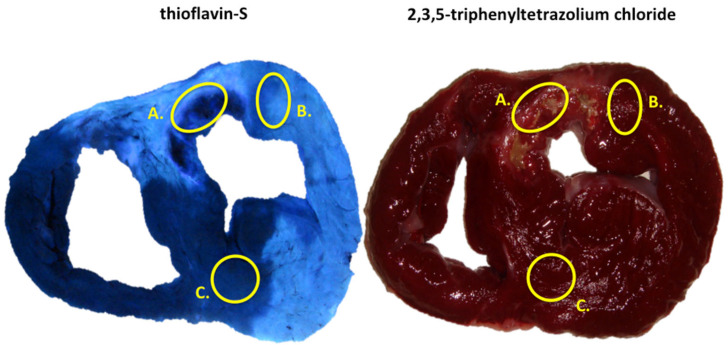
Macroscopic study of myocardial hearts obtained from the swine model. Samples were stained with thioflavin-S (T-S, **left**) and 2,3,5-triphenyltetrazolium chloride (TTZ, **right**). The infarct area (A) was defined as the myocardium located in the area at risk that did not stain with triphenyltetrazolium (thioflavin-S+ and triphenyltetrazolium−). The adjacent area (B) was delimited as the region located in the area at risk that stained with triphenyltetrazolium (thioflavin-S+ and triphenyltetrazolium+). The remote area (C) was the region outside the area at risk (thioflavin-S− and triphenyltetrazolium+).

Immediately after heart excision, the left ventricle was sectioned into 5 mm thick short-axis slices. Firstly, to determine the area at risk, each slide was viewed under ultraviolet light and photographed. Light blue represents thioflavin-S myocardial uptake after infusion through the LAD, while dark blue indicates a lack of LAD perfusion. Next, slices were incubated into 2% 2,3,5-triphenyltetrazolium chloride solution (Sigma Aldrich, St. Louis, MO, USA) at 37 °C for 20 min, viewed under room light and photographed to determine the infarct area [23,26,33] (Figure 5).

The infarct, adjacent, and remote areas within the left ventricle were defined in all short-axis slices. The infarct area was regarded as myocardium located in the area at risk not stained with triphenyltetrazolium (thioflavin-S+ and triphenyltetrazolium−). The adjacent area was defined as the region located in the area at risk stained with triphenyltetrazolium (thioflavin-S+ and triphenyltetrazolium+). The remote area was the region outside the area at risk (thioflavin-S− and triphenyltetrazolium+) [23,26,33] (Figure 5).

Images were digitalized, and manual quantification of all short-axis slices was performed offline by an experienced observer unaware of the experimental protocol applied. The software package MATLAB 6.5 (MathWorks, Natick, MA, USA) was utilized. A ruler was photographed beside myocardial slices in all images and was used as a reference for measurements [33]. LAD-perfused area, infarct extension and myocardial wall thickness of the infarct, adjacent, and remote areas were quantified.

### 4.4. Blood Samples

Blood samples obtained from a multipurpose catheter placed in the coronary sinus were taken at different timepoints of the study protocol: (i) baseline (after surgical procedure and before ischemia induction via angioplasty balloon inflation); (ii) during ischemia (5 min and 75 min after balloon inflation); (iii) after reperfusion (2 h and 48 h after balloon deflation) (Figure 4). In all cases, the coronary catheter location was confirmed by contrast injection.

The coronary serum was isolated by centrifuging blood samples at 2500 rpm for 15 min and stored immediately at −80 °C until further analyses were performed.

### 4.5. Metabolites from the Coronary Sinus

Serial serum samples were taken from the five experimental groups in coronary serum obtained from coronary sinus blood samples. All groups were assayed for glucose, lactate, pyruvate, and NEFA levels.

Glucose and lactate levels were obtained using an ABL800 FLEX blood gas analyzer (Radiometer Medical ApS, Brønshøj, Denmark). Pyruvate and NEFA levels were determined using enzymatic analysis. Absorbance was measured at 340 nm and 546 nm, respectively, using SPECTRAmax Plus 384 equipment (Molecular Devices, San Jose, CA, USA) and the data were processed by GraphPad Prism 6.0. software (Boston, MA, USA). Results are expressed as mg/dL or mM.

### 4.6. RNA Isolation and Quantitative Real-Time Polymerase Chain Reaction

mRNA expression of specific genes of the main myocardial metabolism regulators (PGC1-α, PPARα, ERRα, GLUT-1 and GLUT-4) was assessed in myocardial samples obtained from the infarcted, adjacent, and remote areas in the three experimental groups.

RNA was extracted using an RNeasy Plus Mini Kit (QIAGEN GmbH, Hilden, Germany) according to the manufacturer’s instructions. Gene expression was determined by real time polymerase chain reaction using a 7900HT Fast Real-Time Polymerase Chain Reaction System (Applied Biosystems, ThermoFisher Scientific, Waltham, MA, USA). The values of the threshold cycle (Ct) were determined and normalized to the 18S ribosomal RNA housekeeping gene [34]. We used specific primers pre-designed by Applied Biosystems (ThermoFisher Scientific, Waltham, MA, USA) as specified in Table 2. The fold change in gene expression from the control group was calculated using the 2−ΔΔCt method.

### 4.7. Statistical Analysis

Continuous variables were expressed as mean ± SD. One-way ANOVA analysis was used for comparisons and two-tailed *p*-values less than 0.05 were considered statistically significant. SPSS 19.0 (SPSS, Inc., Chicago, IL, USA) was used throughout.

## 5. Conclusions

Our results show that the mRNA expression of genes involved in FA metabolism heightens during the acute phases post MI, whereas this regulation decreases in the chronic phases. Regarding glucose transporters, a sustained increase in their transcriptomic expression is observed along the reperfusion process. These changes in gene expression parallel the dynamic levels of metabolites in the coronary sinus following MI.

## Figures and Tables

**Table 1:** Macroscopic analysis of the hearts isolated from the animals submitted to myocardial infarction.

	No Reperfusion	1-Min Reperfusion	1-Week Reperfusion	1-Month Reperfusion
LAD-perfused area (% of LV)	54 ± 22	63 ± 10	72 ± 8	66 ± 10
Infarct area (% of LV)	0 ± 0	0 ± 0	28 ± 8	15 ± 4
Myocardial wall thickness:				
Infarct area (mm)	-	-	10 ± 3	10 ± 3
Adjacent area (mm)	11 ± 2	11 ± 2	11 ± 3	11 ± 4
Remote area (mm)	12 ± 4	12 ± 3	11 ± 3	12 ± 4

Data were expressed as mean ± SD. Abbreviations. LAD: Left anterior descending; LV: Left ventricle.

**Table 2:** References of the primers utilized in this study.

Protein	Gene	Reference
PGC1-α	PPARGC1A	Ss0339114_u1
PPARα	PPARA	Ss03380164_u1
ERRα	ESRRA	Ss04246410_m1
GLUT-1	SLC7A1	Ss03374747_s1
GLUT-4	SLC2A4	Ss03373325_g1

Abbreviations. ERRα: Estrogen-related receptors-α; GLUT-1: Glucose transporter 1; GLUT-4: Glucose transporter 4; PGC1-α: Peroxisome proliferator-activated receptor-γ coactivator-1α; PPARα: Peroxisome proliferator-activated receptor-α.

## Data Availability

The raw data supporting the conclusions of this article will be made available by the authors on request.

## Abbreviations

The following abbreviations are used in this manuscript:

ERRα	Estrogen-related receptor-α
FA	Fatty acids
GLUT	Glucose transporter
LAD	Left anterior descending
MI	Myocardial infarction
NEFA	Non-esterified fatty acids
PGC-1α	Peroxisome proliferator-activated receptor-γ coactivator-1α
PPARα	Peroxisome proliferator-activated receptor-α

## References

[1] Bornstein M.R., Tian R., Arany Z. (2024). Human cardiac metabolism. Cell Metab..

[2] Wang H., Liu X., Zhou Q., Liu L., Jia Z., Qi Y., Xu F., Zhang Y. (2023). Current status and emerging trends of cardiac metabolism from the past 20 years: A bibliometric study. Heliyon.

[3] Gibb A.A., Hill B.G. (2018). Metabolic Coordination of Physiological and Pathological Cardiac Remodeling. Circ. Res..

[4] Stanley W.C., Recchia F.A., Lopaschuk G.D. (2005). Myocardial Substrate Metabolism in the Normal and Failing Heart. Physiol. Rev. Am. Physiol. Soc..

[5] Shao D., Tian R. (2015). Glucose Transporters in Cardiac Metabolism and Hypertrophy. Compr. Physiol..

[6] Wang T., Wang J., Hu X., Huang X.J., Chen G.X. (2020). Current understanding of glucose transporter 4 expression and functional mechanisms. World J. Biol. Chem..

[7] Di W., Lv J., Jiang S., Lu C., Yang Z., Ma Z., Hu W., Yang Y., Xu B. (2018). PGC-1: The Energetic Regulator in Cardiac Metabolism. Curr. Issues Mol. Biol..

[8] Aggarwal R., Potel K.N., McFalls E.O., Butterick T.A., Kelly R.F. (2022). Novel Therapeutic Approaches Enhance PGC1-alpha to Reduce Oxidant Stress-Inflammatory Signaling and Improve Functional Recovery in Hibernating Myocardium. Antioxidants.

[9] Cheng C.F., Ku H.C., Lin H. (2018). PGC-1α as a Pivotal Factor in Lipid and Metabolic Regulation. Int. J. Mol. Sci..

[10] Dorn G.W., Vega R.B., Kelly D.P. (2015). Mitochondrial biogenesis and dynamics in the developing and diseased heart. Genes Dev..

[11] Tian H., Zhao X., Zhang Y., Xia Z. (2023). Abnormalities of glucose and lipid metabolism in myocardial ischemia-reperfusion injury. Biomed. Pharmacother..

[12] Huss J.M., Imahashi K., Dufour C.R., Weinheimer C.J., Courtois M., Kovacs A., Giguère V., Murphy E., Kelly D.P. (2007). The nuclear receptor ERRalpha is required for the bioenergetic and functional adaptation to cardiac pressure overload. Cell Metab..

[13] Arany Z., Novikov M., Chin S., Ma Y., Rosenzweig A., Spiegelman B.M. (2006). Transverse aortic constriction leads to accelerated heart failure in mice lacking PPAR-gamma coactivator 1alpha. Proc. Natl. Acad. Sci. USA.

[14] Sihag S., Cresci S., Li A.Y., Sucharov C.C., Lehman J.J. (2009). PGC-1alpha and ERRalpha target gene downregulation is a signature of the failing human heart. J. Mol. Cell. Cardiol..

[15] Lu Z., Xu X., Hu X., Fassett J., Zhu G., Tao Y., Li J., Huang Y., Zhang P., Zhao B. (2010). PGC-1 alpha regulates expression of myocardial mitochondrial antioxidants and myocardial oxidative stress after chronic systolic overload. Antioxid. Redox Signal..

[16] Oka S., Alcendor R., Zhai P., Park J.Y., Shao D., Cho J., Yamamoto T., Tian B., Sadoshima J. (2011). PPARα-Sirt1 complex mediates cardiac hypertrophy and failure through suppression of the ERR transcriptional pathway. Cell Metab..

[17] Ibanez B., Heusch G., Ovize M., Van de Werf F. (2015). Evolving therapies for myocardial ischemia/reperfusion injury. J. Am. Coll. Cardiol..

[18] Murashige D., Jang C., Neinast M., Edwards J.J., Cowan A., Hyman M.C., Rabinowitz J.D., Frankel D.S., Arany Z. (2020). Comprehensive quantification of fuel use by the failing and nonfailing human heart. Science.

[19] Dong S., Qian L., Cheng Z., Chen C., Wang K., Hu S., Zhang X., Wu T. (2021). Lactate and Myocardiac Energy Metabolism. Front. Physiol..

[20] Havmoeller R., Reinier K., Teodorescu C., Ahmadi N., Kwok D., Uy-Evanado A., Chen Y.D., Rotter J.I., Gunson K., Jui J. (2014). Elevated plasma free fatty acids are associated with sudden death: A prospective community-based evaluation at the time of cardiac arrest. Heart Rhythm.

[21] Lopaschuk G.D., Ussher J.R., Folmes C.D., Jaswal J.S., Stanley W.C. (2010). Myocardial fatty acid metabolism in health and disease. Physiol. Rev..

[22] Zuurbier C.J., Bertrand L., Beauloye C.R., Andreadou I., Ruiz-Meana M., Jespersen N.R., Kula-Alwar D., Prag H.A., Eric-Botker H., Dambrova M. (2020). Cardiac metabolism as a driver and therapeutic target of myocardial infarction. J. Cell. Mol. Med..

[23] Ríos-Navarro C., Hueso L., Miñana G., Núñez J., Ruiz-Saurí A., Sanz M.J., Cànoves J., Chorro F.J., Piqueras L., Bodí V. (2018). Coronary Serum Obtained After Myocardial Infarction Induces Angiogenesis and Microvascular Obstruction Repair. Role of Hypoxia-inducible Factor-1A. Rev. Esp. Cardiol..

[24] Macvanin M., Gluvic Z., Radovanovic J., Essack M., Gao X., Isenovic E.R. (2023). New insights on the cardiovascular effects of IGF-1. Front. Endocrinol..

[25] Brosius F.C., Liu Y., Nguyen N., Sun D., Bartlett J., Schwaiger M. (1997). Persistent myocardial ischemia increases GLUT1 glucose transporter expression in both ischemic and non-ischemic heart regions. J. Mol. Cell. Cardiol..

[26] Hervas A., Ruiz-Sauri A., Gavara J., Monmeneu J.V., de Dios E., Rios-Navarro C., Perez-Sole N., Perez I., Monleon D., Morales J.M. (2016). A Multidisciplinary Assessment of Remote Myocardial Fibrosis After Reperfused Myocardial Infarction in Swine and Patients. J. Cardiovasc. Transl. Res..

[27] Tardy-Cantalupi I., Montessuit C., Papageorgiou I., Remondino-Müller A., Assimacopoulos-Jeannet F., Morel D.R., Lerch R. (1999). Effect of transient ischemia on the expression of glucose transporters GLUT-1 and GLUT-4 in rat myocardium. J. Mol. Cell. Cardiol..

[28] Collins H.E. (2023). Female cardiovascular biology and resilience in the setting of physiological and pathological stress. Redox Biol..

[29] Vijay V., Han T., Moland C.L., Kwekel J.C., Fuscoe J.C., Desai V.G. (2015). Sexual dimorphism in the expression of mitochondria-related genes in rat heart at different ages. PLoS ONE.

[30] Medzikovic L., Azem T., Sun W., Rejali P., Esdin L., Rahman S., Dehghanitafti A., Aryan L., Eghbali M. (2023). Sex differences in therapies against myocardial ischemia-reperfusion injury: From basic science to clinical perspectives. Cells.

[31] Garcia-Bustos V., Sebastian R., Izquierdo M., Rios-Navarro C., Bodí V., Chorro F.J., Ruiz-Sauri A. (2019). Changes in the spatial distribution of the Purkinje network after acute myocardial infarction in the pig. PLoS ONE.

[32] Forteza M.J., Trapero I., Hervas A., de Dios E., Ruiz-Sauri A., Minana G., Bonanad C., Gómez C., Oltra R., Rios-Navarro C. (2018). Apoptosis and Mobilization of Lymphocytes to Cardiac Tissue Is Associated with Myocardial Infarction in a Reperfused Porcine Model and Infarct Size in Post-PCI Patients. Oxidative Med. Cell. Longev..

[33] Hervas A., de Dios E., Forteza M.J., Miñana G., Nuñez J., Ruiz-Sauri A., Bonanad C., Perez-Sole N., Chorro F.J., Bodi V. (2015). Intracoronary infusion of thioflavin-S to study microvascular obstruction in a model of myocardial infarction. Rev. Esp. Cardiol..

[34] Ríos-Navarro C., Gavara J., de Dios E., Pérez-Solé N., Molina-García T., Marcos-Garcés V., Ruiz-Saurí A., Bayés-Genís A., Carrión-Valero F., Chorro F.J. (2024). Effect of serum from patients with ST-segment elevation myocardial infarction on endothelial cells. Rev. Esp. Cardiol..

